# A Case of Undiagnosed Placenta Increta Originating From a Demised Twin in the Second Trimester

**DOI:** 10.1155/crog/1329744

**Published:** 2024-11-29

**Authors:** Anthony Grandelis, Jordan Emont, Brittany Arditi, Noelle Breslin, Tarah Pua

**Affiliations:** Department of Obstetrics and Gynecology, Columbia University, New York, New York 10032, USA

## Abstract

Placenta accreta spectrum (PAS) presents a significant risk of maternal morbidity and mortality, in large part due to the potential for massive hemorrhage at time of delivery. Recently, multiple gestations have been shown to be an independent risk factor for PAS, especially in the setting of other major risk factors. Importantly, antenatal detection of PAS in twin pregnancies has been shown to be suboptimal when compared to singleton pregnancies. Here, we present a case of postpartum hemorrhage and unplanned cesarean hysterectomy due to an undiagnosed placenta increta, which originated from the placenta of a demised twin in the second trimester. This case underscores the importance of thorough prenatal monitoring and evaluation for PAS, especially in multifetal gestations with additional risk factors. It also highlights the need for heightened awareness among healthcare providers to mitigate risks associated with PAS in twin pregnancies. Early detection and multidisciplinary collaboration are crucial in optimizing outcomes for both mothers and infants in such complex obstetric scenarios.

## 1. Introduction

Placenta accreta spectrum (PAS) is a term used to encompass placenta accreta, placenta increta, and placenta percreta. All of these anomalies involve abnormal placental implantation where placental villi adhere directly to the myometrium, into the myometrium, or through the myometrium and into the uterine serosa and surrounding structures, respectively [1]. PAS is a leading cause of maternal morbidity and mortality during pregnancy, largely in part due to the massive hemorrhage that can occur prior to or during delivery. Complications of PAS are most commonly attributed to massive hemorrhage and degree of placental invasion. These complications may include disseminated intravascular coagulation, injury to nearby organs, transfusion-related complications, postoperative thromboembolism, multisystem organ failure, and maternal death. The mainstay of treatment for PAS remains cesarean hysterectomy at the time of delivery, which carries an overall mortality rate of 1.6% [2].

Over the last 50 years, rates of PAS have been increasing, with one study citing a prevalence of 1 in 272 pregnancies [3]. This trend is likely in part due to a change in risk factors, most importantly the increased rate of cesarean deliveries [1]. Of the many risk factors associated with PAS, a history of previous cesarean section and concurrent placenta previa confers the highest risk. Other well-known risk factors include prior uterine surgery, advanced maternal age, and use of assisted reproductive technology [4]. Recently, twin gestations have also been reported to pose an elevated risk for PAS. Several case reports have demonstrated this risk to persist even after spontaneous or medically induced reduction of twin pregnancies [5]. Thus, maintaining a high level of suspicion for PAS in pregnancies that initially started as a multifetal gestation, especially in the setting of additional risk factors, is imperative to reducing the risk of maternal morbidity associated with this condition.

## 2. Case Report

**Figure 1:**
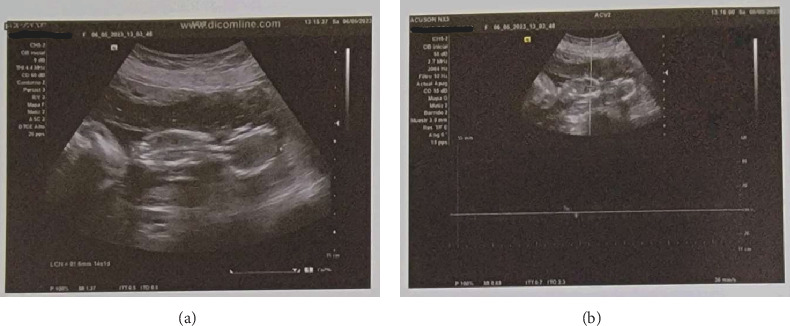
Antenatal ultrasound image showing one twin (b) and absence of fetal heart rate using m-mode (a).

**Figure 2:**
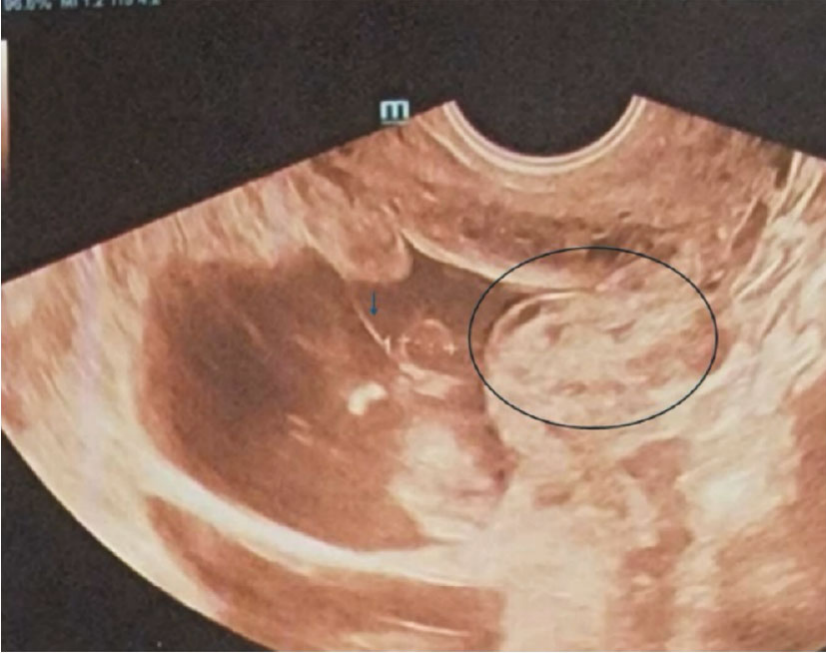
Antenatal ultrasound imaging showing the dividing twin membrane as well as the placenta of the demised twin with an irregular placental interface.

A 43-year-old G3P2002 with a history of two prior low transverse cesarean deliveries presented at 35 weeks and 0 day gestation with vaginal bleeding, contractions, headache, and visual floaters. She had received her prenatal care in the Dominican Republic and had recently immigrated to the United States. Her pregnancy was additionally notable for chronic hypertension and a dichorionic–diamniotic twin pregnancy with a spontaneous reduction at 14w1d. An ultrasound report from the Dominican Republic confirmed dating and revealed a twin gestation with demise of one fetus (Figure 1) and a fundal placenta. There was no comment regarding abnormal placentation or concern for cesarean scar ectopic pregnancy. Additionally, a third trimester ultrasound report confirmed fundal placentation and once again did not report any abnormal findings with regard to placentation. Of note, on retrospective review of antenatal ultrasounds, one image depicted the dividing twin membrane, as well as the placenta of the demised twin with possibly an irregular placental interface (Figure 2). No comment on this possible irregularity was made in the ultrasound report.

On admission, her vitals were normal and bedside ultrasound confirmed a fundal placenta without evidence of placenta previa. There was no bleeding noted on speculum exam, and her cervix was closed. The fetal heart rate tracing was nonreactive, although had good variability. There were several prolonged decelerations over the course of 1–2 h of monitoring, and the tocometer revealed regular contractions every 3–4 min. Her initial laboratory work was notable for normal pre-eclampsia labs and coagulation studies. Given the persistent Category 2 fetal heart rate tracing, reported bleeding, and history of chronic hypertension, urgent delivery was recommended via repeat cesarean section due to concern for placental abruption. Risks of the procedure, including possible hysterectomy, were reviewed with the patient, and informed consent was obtained. The patient was crossed for two units of packed red blood cells (pRBCs) prior to the procedure.

**Figure 3:**
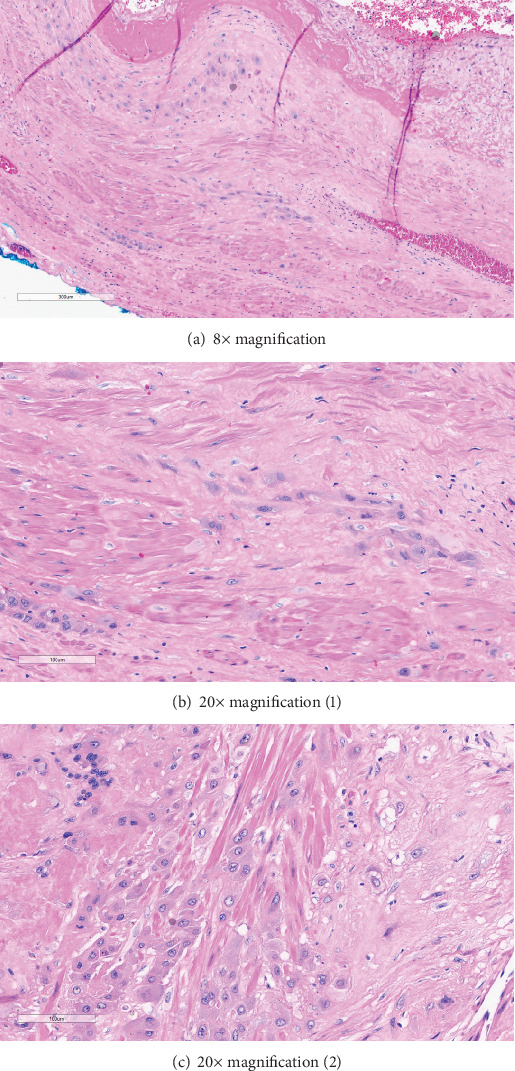
(a–c) Pathology slides confirming diagnosis of placenta increta with trophoblasts permeating through muscle fibers of the deep myometrium. Blue ink denotes the serosa surface of the uterus.

After successful combined spinal–epidural placement, the patient was prepped for surgery and the case was started. Surgical findings were notable for dense adhesive disease from the uterine serosa to the anterior abdominal wall. This adhesive disease required extensive dissection and resulted in significant blood loss even prior to the hysterotomy. After 10 mins of careful dissection, a low transverse hysterotomy was performed and a male neonate was delivered atraumatically, weighing 2385 g with Apgar scores of 4 and 8 at 1 min and 5 min, respectively. Cord gases showed an arterial pH of 7.09 with a base excess of −6.6. The placenta appeared to deliver spontaneously but massive hemorrhage ensued from the lower uterine segment following delivery of the placenta. On manual inspection, a focal area of morbidly adherent placenta was noted in the lower uterine segment below the hysterotomy. The pathology report later confirmed one normal, intact placenta from the liveborn infant and a placenta increta, originating from the placenta of the demised twin (Figures 3(a), 3(b), and 3(c)).

Given the amount of blood loss, the decision to proceed with hysterectomy was made promptly and all team members were notified of undiagnosed PAS and severe postpartum hemorrhage. The massive transfusion protocol was called for and assistance from maternal–fetal medicine and gynecologic oncology was requested. The hysterotomy was closed, and a T midline vertical incision was made just below the umbilicus to aid in visualization. The adhesions from the uterine serosa to the anterior abdominal wall were taken down to allow for exteriorization of the uterus. Attempts at minimizing blood loss during these initial steps were partially achieved through uterine packing with laparotomy sponges and direct manual compression of the uterine arteries.

Once exteriorized, a supracervical hysterectomy proceeded in standard fashion without complication. Due to extensive dissection near the urinary bladder, the bladder was backfilled with methylene blue and no extravasation was noted. Bilateral ureters were evaluated through the abdominal incision and noted to vermiculate at a safe distance from the surgical dissection. In total, the quantitative blood loss was 7500 cc, and the patient received 9 units of pRBCs, 6 units of fresh frozen plasma, and 1 unit of platelets. Her combined spinal–epidural was utilized for pain control through the procedure. She tolerated the procedure well, and there were no apparent intraoperative complications. The patient recovered well in the postpartum period and was discharged on postoperative Day #4.

## 3. Discussion

Multifetal gestations carry an inherently higher risk of maternal and fetal morbidity compared to singleton pregnancies, including increased risk of fetal anomalies, preterm birth, growth restriction, hemorrhage, pre-eclampsia, and need for cesarean delivery [6]. More recently, concern has been raised regarding the potential elevated risk of PAS associated with twin pregnancies. As mentioned previously, complications from PAS are often sequelae of massive hemorrhage and degree of placental invasion, all of which pose a significant risk to maternal morbidity and mortality [2].

There are several hypotheses for the increased risk of PAS seen in twin pregnancies, with the leading theory suggesting that the larger surface area of twin placentas within the lower uterine segment increases the likelihood of abnormal placentation into a previous cesarean scar [6]. It has been suggested that other risk factors for PAS are also commonly associated with multifetal gestations (i.e., advanced maternal age and pregnancies achieved through assistive reproductive technology) and may be confounding factors in this elevated risk. However, in a large retrospective study examining the association between PAS and twin gestations in all live births in California from 2016 to 2017, it was found that twin gestations still conferred an elevated risk for PAS independent of other measured risk factors [4].

Interestingly, the study by Miller et al. showed a lower prevalence of placenta previa in twin gestations affected by PAS and, as a result, a lower likelihood of antenatal detection of PAS in twin pregnancies [4]. This reduced likelihood of antenatal diagnosis was also confirmed by a paper by Shamshirsaz et al., which reported that only 37.5% of twin pregnancies affected by PAS were diagnosed prenatally [7]. These findings are particularly worrisome, as evidenced by the case presented above, for it is well known that early detection and proper delivery planning at an accreta center of excellence are crucial in mitigating maternal and neonatal risks associated with PAS [8]. As might be expected, severe maternal morbidity is higher in twin pregnancies with PAS (compared to singleton gestations), which may be explained by inherent risks associated with multifetal gestations, as well as the decreased rates of antenatal diagnosis of PAS in twin gestations [4]. In the case presented here, undiagnosed PAS resulted in an unanticipated intraoperative diagnosis with subsequent massive hemorrhage. This could most certainly have been mitigated to some extent had there been time for proper preoperative assessment and planning.

Given the outcomes of this case study and other literature regarding PAS in twin pregnancies, prenatal care for twin gestations should include thorough monitoring through ultrasound examinations to assess placental location and monitor for signs of abnormal placentation. This case further highlights that extra vigilance should also be applied to pregnancies where a spontaneous or medically induced reduction of a twin gestation occurred in the first trimester. For patients with multifetal gestations with limited or unknown prenatal care who present to labor and delivery and require urgent delivery, providers should prepare for possible PAS, especially in the setting of other risk factors (particularly higher order cesarean deliveries or placenta previa).

While twin pregnancies inherently present increased challenges in obstetric care, vigilant prenatal awareness of the heightened risk of PAS can contribute to better assessment and outcomes for both mothers and their infants. Collaborative efforts within a multidisciplinary team of healthcare providers will play a pivotal role in navigating the complexities associated with twin pregnancies and reducing the risks associated with PAS disorders in multifetal gestations.

## Data Availability

This manuscript does not include data as part of the case report.
