# Correction to “A Case of Undiagnosed Placenta Increta Originating From a Demised Twin in the Second Trimester”

**DOI:** 10.1155/crog/9786893

**Published:** 2025-09-16

A. Grandelis, J. Emont, B. Arditi, N. Breslin, and T. Pua, “A Case of Undiagnosed Placenta Increta Originating From a Demised Twin in the Second Trimester,” *Case Reports in Obstetrics and Gynecology* 2024 (2024): 1329744, https://doi.org/10.1155/crog/1329744.

In the article, Figure 2 contained identifying information about the patient and has therefore been removed. The results presented in the figure are described in the article:

“An ultrasound report from the Dominican Republic confirmed dating and revealed a twin gestation with demise of one fetus (Figures 1 and 2) and a fundal placenta.”

We apologize for this error.

